# Technical considerations for genotyping multi-allelic copy number variation (CNV), in regions of segmental duplication

**DOI:** 10.1186/1471-2164-15-329

**Published:** 2014-05-01

**Authors:** Stuart Cantsilieris, Patrick S Western, Paul N Baird, Stefan J White

**Affiliations:** Centre for Genetic Diseases, MIMR-PHI Institute of Medical Research, Monash University, 27-31 Wright Street, Clayton, 3168 Victoria Australia; Centre for Eye Research Australia, University of Melbourne, Royal Victorian Eye and Ear Hospital, East Melbourne, Victoria Australia

**Keywords:** Copy number variation, Multi-allelic, *CCL3L1*, Real-time quantitative PCR, Multiplex ligation-dependent probe amplification, Paralogue ratio test

## Abstract

**Background:**

Intrachromosomal segmental duplications provide the substrate for non-allelic homologous recombination, facilitating extensive copy number variation in the human genome. Many multi-copy gene families are embedded within genomic regions with high levels of sequence identity (>95%) and therefore pose considerable analytical challenges. In some cases, the complexity involved in analyzing such regions is largely underestimated. Rapid, cost effective analysis of multi-copy gene regions have typically implemented quantitative approaches, however quantitative data are not an absolute means of certainty. Therefore any technique prone to degrees of measurement error can produce ambiguous results that may lead to spurious associations with complex disease.

**Results:**

In this study we have focused on testing the accuracy and reproducibility of quantitative analysis techniques. With reference to the C-C Chemokine Ligand-3-like-1 (*CCL3L1*) gene, we performed analysis using real-time Quantitative PCR (QPCR), Multiplex Ligation-dependent Probe Amplification (MLPA) and Paralogue Ratio Test (PRT). After controlling for potential outside variables on assay performance, including DNA concentration, quality, preparation and storage conditions, we find that real-time QPCR produces data that does not cluster tightly around copy number integer values, with variation substantially greater than that of the MLPA or PRT systems. We find that the method of rounding real-time QPCR measurements can potentially lead to mis-scoring of copy number genotypes and suggest caution should be exercised in interpreting QPCR data.

**Conclusions:**

We conclude that real-time QPCR is inherently prone to measurement error, even under conditions that would seem favorable for association studies. Our results indicate that potential variability in the physicochemical properties of the DNA samples cannot solely explain the poor performance exhibited by the real-time QPCR systems. We recommend that more robust approaches such as PRT or MLPA should be used to genotype multi-allelic copy number variation in disease association studies and suggest several approaches which can be implemented to ensure the quality of the copy number typing using quantitative methods.

## Background

Structurally complex regions of the human genome contain a wealth of information that is yet to be fully understood within the context of evolution and disease. Here we use the term “complex” to describe genomic regions containing highly duplicated sequence of >95% sequence identity (eg. segmental duplications) demonstrating a range of diploid copies (>3 copy number classes). The C-C Chemokine Ligand-3-like-1 (*CCL3L1)* gene is one such example [1].

The quantitative methods commonly used to genotype multi-allelic copy number polymorphisms (CNPs) are not absolute, therefore accuracy and reproducibly are required to generate robust disease associations [2]. Single or multi locus genotyping methods including Multiplex Ligation-dependent Probe Amplification (MLPA), Paralogue Ratio Test (PRT) and real-time Quantitative PCR (QPCR) are flexible, and cost effective ways to analyze multi-allelic CNPs [3]. Although studies have shown that in some cases these techniques can provide an accurate assessment across a range of diploid copy numbers, their reproducibility can be influenced by a number of factors including assay design, sample quality and operator error.

A challenging aspect of multi-allelic copy number analysis is that the relative difference between copy number classes becomes smaller and more difficult to discriminate as copy number increases [4]. Despite this challenge, CNP analysis should produce data that clusters tightly around integer values [5], unless there is evidence for tissue mosaicism in which different cell types contain different copy numbers [6]. As such, tissue mosaicism can occur over a continuous spectrum (1-99%), and can result in extensive overlap between integer classes resulting in data that seemingly indicates a continuous copy number distribution. However, similar data can also be produced when measurements of non-mosaic tissues are imprecise and unable to discriminate CNVs with a high level of accuracy, potentially leading to erroneous conclusions.

Indeed, differences in these observations made in a number of studies support the conclusion that such measurement error can complicate data interpretation [7] and these effects are clearly evident in studies of the *CCL3L1* gene and disease susceptibility [1, 8, 9]. In some cases, there could be misconceptions surrounding assay validation and/or data interpretation, and if this is coupled with a lack of transparency regarding the distribution of raw unrounded copy number measurements, it may lead to spurious results that are difficult to explain. The *CCL3L1* gene represents a particularly intriguing locus for study, most notably for its implication in HIV AIDS susceptibility [1], but also because this region demonstrates several layers of complexity. In addition to being copy number variable, this locus is highly population stratified and the presence of extreme levels of sequence duplication can complicate assay design. As such due to the presence of the *CC3L3* pseudogene, the analysis of certain *CCL3L1* sequences differs with respect to copy number content and should be considered when attempting to compare methods and studies.

In this study we aim to demonstrate some simple approaches for assessing the quality of genotyping data when using methods such as real-time QPCR, PRT and MLPA for the analysis of multi-allelic copy number. These methods are evaluated by examining the copy number distribution of the *CCL3L1* gene in DNA samples of European ethnicity.

## Methods

### DNA samples

We selected 150 DNA samples from patients of European origin. Genomic DNA was isolated from venous blood leukocytes using a phenol/chloroform extraction procedure as previously described [10]. The concentration and purity of the DNA samples was determined using the NanoDrop Specrophotometer (Thermo Scientific). We considered the genomic DNA samples to be free of contaminants or solvents if the 260/280 ratio resulted in a range between 1.7-2.1 and the 260/230 ratio was >1.5. The samples were collected as part of ongoing eye studies at the Royal Victorian Eye and Ear Hospital (RVEEH), Melbourne. Subjects were given a standard risk factor questionnaire and following clinical examination are considered healthy control individuals. The study was conducted in accordance with the Declaration of Helsinki and according to the National Health and Medical Research Council of Australia’s statement on ethical conduct in research involving humans, revised in 2000. Written informed consent was obtained from all individuals, and ethics approval for the project was provided by the Human Research and Ethics Committee of the Royal Victorian Eye and Ear Hospital (RVEEH), Melbourne.

### Multiplex Ligation-dependent Probe Amplification (MLPA)

Two colour MLPA was conducted as previously described [11]. We designed probes targeting exon 1 and exon 3 of the *CCL3L1* gene listed in Table 1 and these were was normalized against two reference probes known to have a diploid copy number state of two unless there is an obvious phenotype. The reference probes targeted the *CREBBP* and *EP300* genes located on chromosome 16 and 22 respectively (Table 1). The reagents for the MLPA reaction were purchased from Fisher Biotec (Australia). Briefly, 150–250 ng of DNA in a final volume of 5 μl was denatured at 98°C for 5 minutes. After cooling to room temperature, 1.5 μl of probe mix containing *CCL3L1* MLPA probes and two additional reference loci, was combined with 1.5 μl of Hybridization buffer and added to the sample, heat denatured at 95°C for 1 minute followed by hybridization at 60°C for 16 hours. Ligation was performed at 54°C by adding 32 μl of ligation reaction, and after 15 minutes the enzyme was inactivated by heating at 95°C for 5 minutes. Polymerase Chain Reaction (PCR) amplification was carried out under the following conditions: 1 cycle of 98°C for 1 minute; 35 cycles of 95°C 30 seconds, 57°C 30 seconds, 72°C 30 seconds; and 1 cycle of 72°C 20 minutes. The PCR reaction was performed in 25 μl. From each PCR reaction, 1 μl was mixed with 8.9 μl of HiDi formamide and 0.1 μl of ROX500 size standard (Applied Biosystems). Product separation was performed on the ABI 3130 Electrophoresis 16 capillary sequencer (Applied Biosystems). Data Analysis was performed as previously described [12] using the GeneMapper Software (Applied Biosystems) and the peak heights were exported to Microsoft Excel. Each peak height was normalized against the sum of two control loci to generate a ratio, and the normalized ratio was then divided by the median of all ratios for the corresponding peak. These values were ordered from low to high and subgroups corresponding to different DNA copy numbers were calculated to determine the relative differences between the subgroups.

**Table 1:** **MLPA probe sequences used for targeting the** ***CCL3L1*** **gene and two reference loci** ***EP300*** **and** ***CREBBP***

Probe	Left probe	Right probe
***CCL3L1***	GACTCTTGGCTCTGCTGACACTCGAGCCCACATTCCATCACCTGCTCCC	AATCATGCAGGTCTCCACTGCTGCCCTTGCC
**exon 1**		
***CCL3L1***	CCTCCACCTTCCCTCACAGTGT	GTCTGGTGACAACCGAGTGGCT
**exon 3**		
***CREBBP***	CCAGCTAGTGGAATTCAAAACACAATTGGTTCTGTTGGCACA	GGGCAACAGAATGCCACTTCTTTAAGTAACCC
***EP300***	CCAACCTAAGCACTGTTAGTCAGATTGATCCCAGCTCCAT	AGAAAGAGCCTATGCAGCTCTTGGACTACCCTATCA

### Real-time quantitative PCR (QPCR)

Real-time Quantitative PCR was performed according to the TaqMan® Copy Number Assays protocol (Applied Biosystems). We used 20 ng/μL of genomic DNA in a 10 μL reaction, consisting of 5 μL of 2× TaqMan® Genotyping Master Mix, 0.5 μL of RNaseP TaqMan assay 20× working stock, 0.625 μL of *CCL3L1* Forward 10 μM, 0.625 μL of *CCL3L1* Reverse 10 μM, 0.3 μL of *CCL3L1* MGB probe 10 μM and 1 μL DH_2_O. The *CCL3L1* MGB probe sequence is as follows MGB-6FA: TTCGAGGCCCAGCGACCTCA. The PCR primer sequences are as follows CCL3L1 Forward: GGGTCCAGAAATACGTCAGT and CCL3L1 Reverse: CATGTTCCCAAGGCTCAG as previously described [13]. Reactions were performed in duplicate, the cycling times were 1 cycle of 95°C for 10 minutes, and 40 cycles of 95°C for 15 seconds and 60°C for 1 minute. To assess the PCR efficiencies of the test and reference locus, we performed a series of 10-fold serial dilutions for both the *CCL3L1* and RNaseP PCR reactions. We found that the PCR efficiencies were approximately equal, differing by less than 1%. We exported the C_T_ values for both and analyzed them using CopyCaller™ software from Applied Biosystems. CopyCaller™ performs a comparative C_T_ (ΔΔC_T_) relative quantitation analysis whereby the difference between the threshold cycles of the target and reference gene is calculated, the ΔC_T_ of the test sample is compared to a calibrator of known copy number. We chose the calibrator sample which represented two copies of the *CCL3L1* gene as tested by MLPA.

### Paralogue Ratio Test (PRT)

We performed Single Plex Paralogue Ratio Test (PRT) using the method described previously [5]. We used 20 ng/μL of genomic DNA in a 25 μL reaction, comprising of 5 μL of 5× Bioline My Taq Buffer, 2 μL of 10 pmol/μL CCL3C Forward: GGC TAA GAC CCC TTC TAG AG and CCL3C Reverse: AAT CAT GCA GGT CTC CAC T Primer, 1 μL Dimethyl sulfoxide (DMSO), 0.25 μL Bioline My Taq Polymerase and 14.75 μL DH_2_O. The cycling times were 1 cycle of 95°C for 1 minute, 24 cycles of 95°C for 30 seconds, 55°C for 30 seconds and 70°C for 1 minute, followed by 1 cycle of 70°C for 40 minutes. The PCR primers were FAM labeled and for each PCR reaction, 1 μl was mixed with 8.9 μl of HiDi Formamide and 0.1 μl of ROX500 size standard (Applied Biosystems). PCR product separation was performed on the ABI 3130 Electrophoresis 16 capillary sequencer (Applied Biosystems). Data Analysis was performed using the GeneMapper Software (Applied Biosystems) and the peak heights were exported to Microsoft Excel. Copy number was calculated as the ratio of the *CCL3L1* peak height against the reference peak height of the diploid *CCL3* gene.

## Results

### Assay design

**Figure 1:**
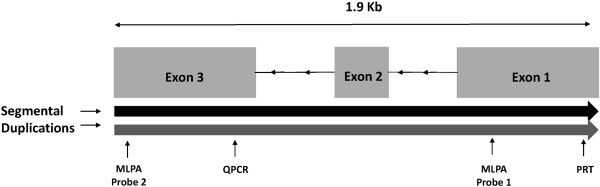
**Schematic representation of the** ***CCL3L1*** **gene located on chromosome 17q12.** Overlapping segmental duplications are indicated by the black and grey arrows and the probe and primer positions for the PRT, real-time QPCR and MLPA assays are indicated by labels.

The *CCL3L1* gene is approximately 1.9 kb in size and resides in a 90 kb segmental duplication on chromosome 17q12 (Figure 1). *CCL3L1* contains 3 exons and shares >95% sequence identity with the *CCL3* gene. As exons 2 and 3 are duplicated in the *CCL3L* pseudogene, a quantitative assay targeting exons 2 or 3 will in some instances, produce 1–2 copies more in comparison to an assay targeting exon 1. The PRT assay previously described by Carpenter *et al.* was specifically designed to avoid targeting the *CCL3L* pseudogene, an approach to only amplify the active gene in disease association studies [5]. In contrast, many studies (reviewed in [7]) using real-time QPCR, have targeted exon 3 also co-amplifying the *CCL3L* pseudogene. The real-time QPCR primers used in this study, have been described previously and target exon 3 [13]. In order to allow a direct comparison between these systems, we leveraged the multiplexing capability of MLPA, and designed two probes targeting exons 1 and 3 of the *CCL3L1* gene (Figure 1). As the real-time QPCR assay targets exon 3 and the PRT assay targets exon 1, we could then compare both techniques to the two individual probe results generated by the MLPA assay.

### Validation of CNV genotyping methods

In order to validate each of the three genotyping methodologies, we compared 12 samples previously identified as having 1, 2, 3 or 4 *CCL3L1* copies (three samples of each copy number class) using the MLPA assay, and compared these with results generated by both the PRT and real-time QPCR systems. We found that the genotype calls between all three methodologies were highly concordant, demonstrating that each assay represented data specific to each exon.

### Assessment of assay performance and separation of integer copy number

**Figure 2:**
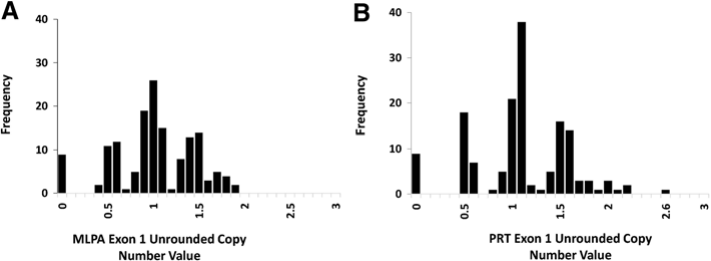
**The distribution of unrounded** ***CCL3L1*** **copy number measurements plotted on a histogram from the PRT and MLPA exon 1 assays. A**. The MLPA assay demonstrates clear clustering, with peaks centered on integer values and gaps between each of the clusters ranging from 0–4 copies. **B**. The PRT assay demonstrates a distribution of 0–5 copies, with peaks clearly centered on integer values.

**Figure 3:**
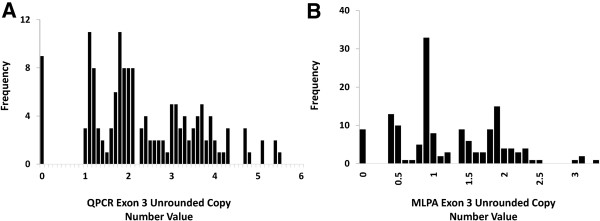
**The distributions of unrounded** ***CCL3L1*** **copy number measurements plotted on a histogram from real-time QPCR and MLPA exon 3 assays. A**. The real-time QPCR assay demonstrates substantial variation around integer values. Without defined gaps between each of the clusters, assignment of whole copy number is performed by rounding the data to the nearest integer. **B**. The MLPA assay demonstrates a distribution of 0–6 copies with peaks centered on integer values. Copy number assignment can be performed by the groups of samples to a particular cluster.

**Figure 4:**
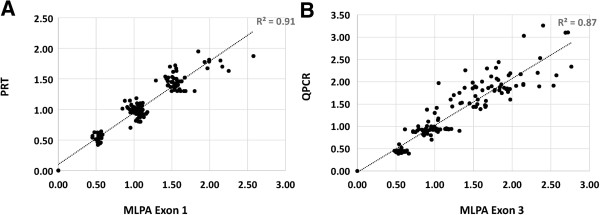
**Comparison of the raw ratio data between MLPA, PRT and real-time QPCR using concordance plots. A**. MLPA and PRT based measurements clearly demonstrates grouping around integer values and excellent correlation, as indicated by the R^2^ value of 0.91. Five samples represent a difference between PRT and MLPA of a single copy, however clustering both PRT and MLPA measurements allows copy number to be unambiguously assigned. **B**. A comparison between MLPA and real-time QPCR demonstrates a greater degree of variation between the two measurements. An almost vertical spread of measurements on the concordance plot demonstrates a poor degree of clustering in the 3–6 copy range between the two assays. The correlation between real-time QPCR and MLPA is high (R^2^ 0.87), suggesting a low level of precision with respect to QPCR.

We genotyped 150 genomic DNA samples using three quantitative assays MLPA, PRT and real-time QPCR. We plotted the raw unrounded copy number estimates on a histogram for all samples to provide an overall assessment on the quality of the data (Figures 2 and 3). Both the PRT and MLPA exon 1 systems demonstrated clear grouping of measures, consistent with integer clustering (Figure 2A and B). Clearly distinguishable from the histogram are 4 distinct clusters, each surrounding a normalized ratio of 0, 0.5, 1.5, and 2 consistent with 0, 1, 2, 3 and 4 diploid *CCL3L1* copies. A direct comparison of copy number genotypes demonstrated 97% (145/150) concordance of copy number calls between the two assays with the exception of five samples within the three to five copy number range differing by only single copy (Figure 4A). Using a concordance plot to examine the degree of correlation between the MLPA and PRT systems, we demonstrated an R^2^ value of 0.91. The high degree of clustering exhibited by the histogram plots of raw unrounded copy number and the high degree of correlation and clustering shown in the concordance plot leaves little doubt as to the assignment of integer copy number (Figure 4A). We next examined the performance of real-time QPCR, with respect to cluster quality and assignment of integer copy number by comparison to data obtained by MLPA in the same 150 DNA samples. As the real-time QPCR and MLPA exon 3 assays amplify the *CCL3L* pseudogene, the range and frequency of integer copy number extends from 0–6 copies, with a higher frequency of 4 and 5 copy genotypes than would be expected if only exon 1 was amplified. Contrary to the data we observed with the MLPA system, the real-time QPCR assay performed quite poorly over a large sample range (Figure 3A). We found that overall, real-time QPCR produced a continuous spread of copy number values rather than discrete clusters (Figure 3A). Interestingly, real-time QPCR appeared to demonstrate a far more consistent clustering around 1 and 2 copy number classes, compared to reliably clustering the difference between 2, 3, 4 and higher copy number classes. As we could not adequately infer copy number genotype based on the clustering of unrounded measures, the 2^-ΔΔCT^ values for the real-time QPCR assay were rounded to the nearest copy number integer. We achieved 81% (121/150) concordance with genotypes obtained with MLPA, with the highest discrepancies present in the 3 from 4 copy range (Table 2). Interestingly, when we performed the concordance plot between the two assays, the R^2^ between the two assays was higher than expected (R^2^ 0.87), yet the overall cluster quality was quite poor (Figure 4B), suggesting that real-time QPCR has a reasonable level of accuracy but exhibits poor precision.

**Table 2:** **CNV genotyping calls from three independent techniques, QPCR, PRT and MLPA, when analyzing the** ***CCL3L1*** **gene**

	***CCL3L1*** exon 1		***CCL3L1*** exon 3	
C#	PRT	MLPA	MLPA	QPCR
0 copies	9	9	9	9
1 copy	25	25	24	26
2 copies	67	67	50	53
3 copies	39	43	22	28
4 copies	9	6	35	25
5 copies	1	0	6	7
6 copies			4	2

## Discussion

Measurement error has the power to drastically influence disease association studies, and this is particularly evident when performing analysis of multi-allelic CNPs. Studies have shown that even a small degree of differential bias, applied systematically to large case/control data sets, is likely to result in spurious association between multi-allelic CNPs and disease [2, 5] Our results demonstrate quite conclusively that real-time QPCR is prone to measurement error and performs poorly when applied to the *CCL3L1* multi-allelic CNP region, when compared to alternative techniques such as MLPA or PRT.

The performance of real-time QPCR has previously been assessed for genotyping multi-allelic CNP regions such as the *CCL3L1* gene [14] and the Beta-defensin gene cluster [2, 15, 16]. In each case, two main conclusions concerning the results have been reported 1. There is a lack of clear integer clustering 2. There are discrepancies between some copy number calls when compared to alternative approaches. Both of these findings are supported by the data presented here. Previous studies have attempted to compare the performance of *CCL3L1* genotyping using the real-time QPCR and PRT systems [14]. However as the assays targeted different exons, the presence of a pseudogene excluded a more direct comparison of the techniques. Additionally, the cases and controls used in the study by Field *et al*. were extracted using different techniques potentially introducing variation in template quality as a contributor to the poor performance of real-time QPCR.

Several studies have suggested that the biological properties of the DNA including protein content, shearing, methods of extraction and storage conditions are potential contributors to the poor performance of real-time QPCR for analyzing multi-allelic CNPs [2, 14, 15, 17]. Additionally, it has been suggested that real-time QPCR will perform comparably to PRT if the concentration of DNA samples is normalized to a uniform concentration, and the efficiency between the test and reference loci differ by less than 5% [18]. With knowledge of these potential variables on assay performance, we felt that it would be unfair to perform a method comparison on DNA samples that were subject to such limitations. Therefore, we reasoned that if DNA quality is crucial to the success of real-time QPCR, then using high quality input DNA samples of similar concentration, generated using a single technique, and stored under uniform conditions, should produce high quality data. However, the results we present here do not support the conclusion that sample quality or PCR efficiency are the only reason for the variability observed with the real-time QPCR system, and thus we present an alternative hypothesis.

A question that has not been expressly raised when conducting disease association studies using real-time QPCR has been; what is the required number of technical replicates to generate reproducible data using an appropriate standard deviation? We contend that, due to the dynamic nature of multi-allelic CNPs a standardized number of technical replicates (for example 2–3), is inappropriate when the difference between copy number integers is not static. Evidence to support this was reported by Weaver *et al.* 2010 where using a standard deviation of 0.16, and false positive and false negative rate of 2.5%, would theoretically require a minimum of 4 replicates to distinguish 1 copy from 2 copies, 6 replicates to distinguish 2 copies from 3 copies, 11 replicates to distinguish 3 copies from 4 copies and 17 replicates to distinguish 4 copies from 5 copies [19]. We summarized some of the larger studies which have utilized real-time QPCR to analyze the *CCL3L1* gene and the Beta defensins for disease association, and find that in all but one case, two technical replicates have been used to conduct the association study (Table 3). In the study presented here we also performed a duplicate testing approach to real-time QPCR, so that our data could be compared to previous work. However, under this model, it would appear that the use of real-time QPCR is questionable for analyzing the *CCL3L1* gene which commonly varies between 0–4 copies per diploid genome [5] and totally inappropriate for beta defensins given the copy number range is much higher, commonly between 2–7 copies [20].

**Table 3:** **Reported number of technical replicates used in real-time QPCR studies for disease association**

Studies	Gene	Disease association	Technical replicates performed
Gonzalez *et al.*[1]	*CCL3L1*	HIV related Outcomes	2
Urban *et al.*[9]	*CCL3L1*	HIV related Outcomes	2
Bhattacharya *et al.*[8]	*CCL3L1*	HIV related Outcomes	3
Field *et al.*[14]	*CCL3L1*	T1 Diabetes	2
Mamtani *et al.*[21]	*CCL3L1*	Systemic Lupus Erythematosus	2
Fellerman *et al.*[22]	Beta-defensin	Crohn’s Disease	3
Bentley *et al.*[23]	Beta-defensin	Crohn’s Disease	2
Mehlotra *et al.*[24]	Beta-defensin	HIV related Outcomes	2

Previous studies have highlighted the critical importance of implementing copy number reference samples, plotting the raw unrounded copy number measurement data and observing the clustering around integer values in disease association studies [2, 5, 25]. We also demonstrate that assay validation should not consist solely in genotyping positive controls as real-time QPCR performed comparably to PRT and MLPA when a small number of positive controls were genotyped. Our data shows that because the variance for real-time QPCR measurements surrounding integer values is substantially greater than that of the PRT or MLPA systems, only by plotting and ordering the raw unrounded measurements from a larger sample size on a histogram can the short-comings of QPCR be identified. It is when a smaller sample size is utilized that the results can potentially be misleading. We would like to point out that this can be done by genotyping as few as 40 test samples, well before an entire cohort is genotyped. Therefore, we believe the use of positive controls should be strictly limited to assessing the ability of an assay to detect copy number variation, and then calibrating inter-run variation.

In some cases, studies using real-time QPCR have systematically removed uncertain data that fails to cluster around integer values in an attempt to reduce the impact of measurement error [26, 27]. However, calling samples with only a high degree of certainty may result in bias, as the failure to cluster is unlikely to be independent of genotype [28]. A more robust approach using real-time QPCR would be to firstly; perform a sufficient number of replicates as dictated by the copy number content of the given region; secondly repeat samples in which the inference of the most likely copy number has extended error bars, utilize the z-scores to highlight data with low confidence [29]; and thirdly, implement a statistical model such as CNVtools which specifically controls for differential bias in the case/control analysis while also allowing one to input data as probability weighted CNV scores as well as integers [28].

One of the more troubling aspects regarding the use of real-time QPCR in our study is the failure to form integer clusters on a sample set of high quality DNA of similar concentration. This suggests that real-time QPCR is inherently prone to measurement error. Moreover this error may be exacerbated by varying sample quality in large case/control cohorts potentially leading to false positive associations. However, it is also noteworthy that real-time QPCR is not substantially incorrect in its assessment of copy number genotype, as demonstrated in Table 2. Our data shows that even though under optimal conditions the variance of real-time QPCR is considerable across large sample numbers, however it still may have a place as purely a confirmatory assay for sequencing projects or MLPA and PRT data sets.

The consistent level of clustering observed by both the PRT and MLPA systems performed without replicates or repeat testing is unequivocal evidence of their accuracy and reliability on a large scale. Additionally, we have not fully utilized the multiplexing capacity of MLPA [11] or Triplex PRT [5] which would further improve the copy number typing of these systems. One important aspect to consider is that combining measurements from two independent techniques, such as PRT and MLPA, is very powerful with respect to accuracy and reproducibility. We found that MLPA and PRT calls differed by a single copy in five samples, notably in the 4–5 copy range. However, by combining the two techniques using a concordance plot we were able to unambiguously assign copy number to these samples (Figure 4A).

We argue that many multi-allelic CNP regions have been largely underestimated with regard to the complexity of analysis, as evidenced by the large number of contradictory evidence concerning disease association studies (reviewed in [7]). There also appears to be a fundamental need to understand how these techniques work, along with which variables can impact the reproducibility of the assay. We also highlight that real-time QPCR appears to be satisfactory for detecting differences between 1 and 2 copies, thus may be appropriate for the detection of di-allelic CNPs where the differences between the integers are at least 2-fold. Recently, water in oil digital droplet PCR (ddPCR) has been described for genotyping multi-allelic loci with an impressive level of reproducibility and accuracy [30, 31]. While this technology has not yet been widely implemented for association studies, it is likely that ddPCR will resolve a number of issues regarding measurement error in large cohorts. The development of a low cost genotyping assay that can provide accurate copy number content, and also possesses the ability to discriminate sequence content, including smaller insertion/deletions and paralogue specific variation, is required to fully understand the complex nature of multi-copy genomic regions. Recent work combining molecular inversion probes (MIPs) together with massively parallel DNA sequencing [32] appears perfectly suited for this type of analysis and will bridge the gap between locus specific quantitative measures and whole genome sequencing technologies. Although MIPs have immense promise for accurately genotyping multi-allelic CNPs such as *CCL3L1*, it is noteworthy that MIP technology is heavily reliant on high quality reference sequences and availability of singly unique nucleotides (SUNs) to accurately distinguish gene family paralogs. Therefore at present MIP- based genotyping cannot be universally applied to all multi-allelic CNP regions.

## Conclusions

In conclusion the data presented here clearly demonstrates that real-time QPCR is prone to measurement error when applied to genotyping the *CCL3L1* multi-allelic CNP region. We recommend that real-time QPCR data be treated with a high degree of caution and suggest that if it is used for the analysis of multi-allelic CNPs, that it is accompanied by independent genotyping measures such as MLPA or PRT. With reference to quantitative measures, ddPCR technology, MIPs or MLPA and PRT, represent far more reliable and robust approaches for accurately resolving multi-allelic CNPs and should be used in preference if possible.

### Availability of supporting data

The data supporting the results of this article are included within the article.

## Authors’ informations

Paul N Baird and Stefan J White are recognized as joint senior authors.
